# (*E*)-*N*′-(2,4-Dihy­droxy­benzyl­idene)-4-nitro­benzohydrazide

**DOI:** 10.1107/S1600536811002224

**Published:** 2011-01-22

**Authors:** Nooraziah Mohd Lair, Hamid Khaledi, Hapipah Mohd Ali

**Affiliations:** a Department of Chemistry, University of Malaya, 50603 Kuala Lumpur, Malaysia

## Abstract

The title compound, C_14_H_11_N_3_O_5_, is essentially planar, with an r.m.s. deviation for the non-H atoms of 0.0832 (3) Å. In the crystal, O—H⋯O and N—H⋯O hydrogen bonds link adjacent mol­ecules into layers parallel to (101). These layers are further connected into a three-dimensional network *via* C—H⋯O inter­actions. In addition, a π–π inter­action occurs between the aromatic rings [centroid–centroid distance = 3.5425 (8) Å]. An intra­molecular O—H⋯N hydrogen bond is also observed.

## Related literature

For related structures, see: Han & Zhao (2010 ▶); Mohd Lair *et al.* (2009 ▶); Raj *et al.* (2008 ▶).
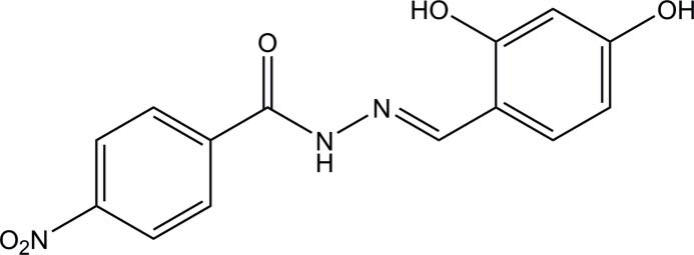


## Experimental

### 

#### Crystal data

C_14_H_11_N_3_O_5_
*M* *_r_* = 301.26
Monoclinic, 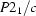

*a* = 8.0248 (1) Å
*b* = 12.5674 (2) Å
*c* = 12.8770 (2) Å
β = 96.732 (1)°
*V* = 1289.70 (3) Å^3^
*Z* = 4
Mo *K*α radiation
μ = 0.12 mm^−1^
*T* = 100 K
0.21 × 0.15 × 0.08 mm

#### Data collection

Bruker APEXII CCD diffractometer
Absorption correction: multi-scan (*SADABS*; Sheldrick, 1996 ▶) *T*_min_ = 0.975, *T*_max_ = 0.990
10373 measured reflections
2398 independent reflections
2004 reflections with *I* > 2σ(*I*)
*R*_int_ = 0.027

#### Refinement

*R*[*F* ^2^ > 2σ(*F* ^2^)] = 0.034
*wR*(*F* ^2^) = 0.099
*S* = 1.05
2398 reflections
208 parameters
3 restraints
H atoms treated by a mixture of independent and constrained refinement
Δρ_max_ = 0.22 e Å^−3^
Δρ_min_ = −0.27 e Å^−3^

### 

Data collection: *APEX2* (Bruker, 2007 ▶); cell refinement: *SAINT* (Bruker, 2007 ▶); data reduction: *SAINT*; program(s) used to solve structure: *SHELXS97* (Sheldrick, 2008 ▶); program(s) used to refine structure: *SHELXL97* (Sheldrick, 2008 ▶); molecular graphics: *X-SEED* (Barbour, 2001 ▶); software used to prepare material for publication: *SHELXL97* and *publCIF* (Westrip, 2010 ▶).

## Supplementary Material

Crystal structure: contains datablocks I, global. DOI: 10.1107/S1600536811002224/is2667sup1.cif

Structure factors: contains datablocks I. DOI: 10.1107/S1600536811002224/is2667Isup2.hkl

Additional supplementary materials:  crystallographic information; 3D view; checkCIF report

## Figures and Tables

**Table 1:** Hydrogen-bond geometry (Å, °)

*D*—H⋯*A*	*D*—H	H⋯*A*	*D*⋯*A*	*D*—H⋯*A*
O1—H1⋯N1	0.86 (2)	1.93 (2)	2.6818 (15)	146 (2)
O2—H2*A*⋯O3^i^	0.84 (1)	1.84 (2)	2.6759 (14)	173 (2)
N2—H2*B*⋯O5^ii^	0.87 (1)	2.28 (1)	3.0606 (16)	150 (1)
C2—H2⋯O3^i^	0.95	2.47	3.1730 (17)	131
C4—H4⋯O4^iii^	0.95	2.54	3.3428 (17)	143
C7—H7⋯O5^ii^	0.95	2.40	3.2082 (17)	143
C10—H10⋯O4^ii^	0.95	2.52	3.3627 (18)	147

Symmetry codes: (i) 
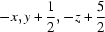
; (ii) 
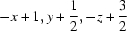
; (iii) 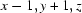.

**Fig. 1.**
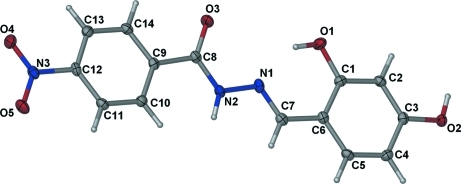
Displacement ellipsoid plot of the title compound at the 50% probability level. Hydrogen atoms are drawn as spheres of arbitrary radius

## supplementary crystallographic information

### Comment

The title compound is the product of the condensation reaction of 4-nitrobenzohydrazide and 4-hydroxysalicylaldehyde. In agreement with the structures of similar benzoylhydrazones (Han & Zhao, 2010; Mohd Lair *et al.*, 2009; Raj *et al.*, 2008), the molecular structure of the present molecule is almost planar, the r.m.s. deviation for the non-H atoms being 0.0832 Å. The crystal structure is stabilized by O—H···O, N—H···O and C—H···O intermolecular and also O—H···N intramolecular hydrogen bonding. Moreover, a π–π interaction occurs between the aromatic rings of pairs of molecules related by symmetry -*x*, -*y* + 1, -*z* + 2 with centroid-centroid separation of 3.5425 (8) Å.

### Experimental

A mixture of 4-nitrobenzohydrazide (0.54 g, 3 mmol) and 4-hydroxysalicylaldehyde (0.39 g, 3 mmol) in ethanol (50 ml) and in the presence of a few drops of acetic acid was refluxed for 5 hr. The solution was then left at room temperature. The crystals of the title compound were obtained in a few days.

### Refinement

The carbon-bound H atoms were placed at calculated positions (C—H = 0.95 Å) and treated as riding on their parent carbon atoms. The nitrogen- and oxygen-bound H atoms were located in a difference map and refined as free atoms, with N—H and O—H distances restrained to 0.88 (2) and 0.84 (2) Å, respectively. *U*_iso_(H) values were set to 1.2–1.5 *U*_eq_(carrier atom).

### Crystal data

**Table:**

C_14_H_11_N_3_O_5_	*F*(000) = 624
*M**_r_* = 301.26	*D*_x_ = 1.552 Mg m^−^^3^
Monoclinic, *P*2_1_/*c*	Mo *K*α radiation, λ = 0.71073 Å
Hall symbol: -P 2ybc	Cell parameters from 3471 reflections
*a* = 8.0248 (1) Å	θ = 3.2–30.4°
*b* = 12.5674 (2) Å	µ = 0.12 mm^−^^1^
*c* = 12.8770 (2) Å	*T* = 100 K
β = 96.732 (1)°	Block, orange
*V* = 1289.70 (3) Å^3^	0.21 × 0.15 × 0.08 mm
*Z* = 4	

### Data collection

**Table:**

Bruker APEXII CCD diffractometer	2398 independent reflections
Radiation source: fine-focus sealed tube	2004 reflections with *I* > 2σ(*I*)
graphite	*R*_int_ = 0.027
φ and ω scans	θ_max_ = 25.5°, θ_min_ = 2.3°
Absorption correction: multi-scan (*SADABS*; Sheldrick, 1996)	*h* = −9→9
*T*_min_ = 0.975, *T*_max_ = 0.990	*k* = −15→12
10373 measured reflections	*l* = −15→15

### Refinement

**Table:**

Refinement on *F*^2^	Primary atom site location: structure-invariant direct methods
Least-squares matrix: full	Secondary atom site location: difference Fourier map
*R*[*F*^2^ > 2σ(*F*^2^)] = 0.034	Hydrogen site location: inferred from neighbouring sites
*wR*(*F*^2^) = 0.099	H atoms treated by a mixture of independent and constrained refinement
*S* = 1.05	*w* = 1/[σ^2^(*F*_o_^2^) + (0.0584*P*)^2^ + 0.2741*P*] where *P* = (*F*_o_^2^ + 2*F*_c_^2^)/3
2398 reflections	(Δ/σ)_max_ = 0.001
208 parameters	Δρ_max_ = 0.22 e Å^−^^3^
3 restraints	Δρ_min_ = −0.27 e Å^−^^3^

### Special details

**Table:**

Geometry. All e.s.d.'s (except the e.s.d. in the dihedral angle between two l.s. planes) are estimated using the full covariance matrix. The cell e.s.d.'s are taken into account individually in the estimation of e.s.d.'s in distances, angles and torsion angles; correlations between e.s.d.'s in cell parameters are only used when they are defined by crystal symmetry. An approximate (isotropic) treatment of cell e.s.d.'s is used for estimating e.s.d.'s involving l.s. planes.
Refinement. Refinement of *F*^2^ against ALL reflections. The weighted *R*-factor *wR* and goodness of fit *S* are based on *F*^2^, conventional *R*-factors *R* are based on *F*, with *F* set to zero for negative *F*^2^. The threshold expression of *F*^2^ > σ(*F*^2^) is used only for calculating *R*-factors(gt) *etc*. and is not relevant to the choice of reflections for refinement. *R*-factors based on *F*^2^ are statistically about twice as large as those based on *F*, and *R*- factors based on ALL data will be even larger.

### Fractional atomic coordinates and isotropic or equivalent isotropic displacement parameters (Å^2^)

**Table:**

	*x*	*y*	*z*	*U*_iso_*/*U*_eq_	
O1	0.01817 (13)	0.64009 (9)	1.21087 (8)	0.0250 (3)	
H1	0.073 (2)	0.5985 (14)	1.1740 (14)	0.038*	
O2	−0.25926 (12)	0.97321 (8)	1.19160 (8)	0.0200 (2)	
H2A	−0.271 (2)	0.9547 (14)	1.2529 (12)	0.030*	
O3	0.31162 (13)	0.40161 (8)	1.11922 (8)	0.0232 (3)	
O4	0.70514 (13)	0.04460 (8)	0.83143 (8)	0.0287 (3)	
O5	0.69197 (12)	0.15345 (9)	0.70111 (8)	0.0248 (3)	
N1	0.15626 (13)	0.57805 (9)	1.04095 (9)	0.0180 (3)	
N2	0.23932 (14)	0.50897 (9)	0.98041 (9)	0.0173 (3)	
H2B	0.2412 (19)	0.5289 (12)	0.9163 (11)	0.021*	
N3	0.66607 (14)	0.13011 (10)	0.79089 (9)	0.0193 (3)	
C1	−0.02829 (16)	0.72791 (11)	1.15383 (11)	0.0170 (3)	
C2	−0.11597 (16)	0.80504 (11)	1.20227 (11)	0.0171 (3)	
H2	−0.1393	0.7950	1.2722	0.021*	
C3	−0.16953 (16)	0.89687 (11)	1.14835 (11)	0.0168 (3)	
C4	−0.13465 (16)	0.91253 (11)	1.04548 (11)	0.0182 (3)	
H4	−0.1708	0.9756	1.0088	0.022*	
C5	−0.04773 (16)	0.83605 (11)	0.99795 (11)	0.0178 (3)	
H5	−0.0243	0.8472	0.9282	0.021*	
C6	0.00759 (16)	0.74153 (11)	1.04988 (11)	0.0165 (3)	
C7	0.09681 (16)	0.66335 (11)	0.99533 (11)	0.0178 (3)	
H7	0.1119	0.6752	0.9242	0.021*	
C8	0.31525 (16)	0.42271 (11)	1.02595 (11)	0.0162 (3)	
C9	0.40734 (15)	0.35067 (11)	0.95936 (10)	0.0156 (3)	
C10	0.44714 (16)	0.37683 (11)	0.85999 (11)	0.0171 (3)	
H10	0.4134	0.4436	0.8299	0.020*	
C11	0.53565 (16)	0.30596 (11)	0.80500 (11)	0.0178 (3)	
H11	0.5651	0.3237	0.7378	0.021*	
C12	0.58004 (16)	0.20868 (11)	0.85038 (11)	0.0171 (3)	
C13	0.54399 (16)	0.18076 (11)	0.94917 (11)	0.0183 (3)	
H13	0.5776	0.1138	0.9788	0.022*	
C14	0.45772 (16)	0.25303 (11)	1.00359 (11)	0.0174 (3)	
H14	0.4325	0.2359	1.0719	0.021*	

### Atomic displacement parameters (Å^2^)

**Table:**

	*U*^11^	*U*^22^	*U*^33^	*U*^12^	*U*^13^	*U*^23^
O1	0.0306 (6)	0.0227 (6)	0.0229 (6)	0.0100 (5)	0.0082 (4)	0.0054 (4)
O2	0.0261 (5)	0.0173 (5)	0.0178 (5)	0.0037 (4)	0.0071 (4)	−0.0005 (4)
O3	0.0320 (6)	0.0225 (6)	0.0170 (5)	0.0004 (4)	0.0110 (4)	0.0007 (4)
O4	0.0369 (6)	0.0231 (6)	0.0256 (6)	0.0117 (5)	0.0013 (5)	−0.0019 (5)
O5	0.0252 (5)	0.0326 (6)	0.0179 (5)	0.0014 (5)	0.0076 (4)	−0.0044 (4)
N1	0.0152 (5)	0.0186 (6)	0.0209 (6)	−0.0013 (5)	0.0057 (5)	−0.0055 (5)
N2	0.0182 (6)	0.0190 (6)	0.0156 (6)	0.0005 (5)	0.0063 (5)	−0.0032 (5)
N3	0.0165 (6)	0.0231 (7)	0.0180 (6)	0.0001 (5)	0.0003 (5)	−0.0053 (5)
C1	0.0145 (6)	0.0173 (7)	0.0190 (7)	−0.0017 (5)	0.0007 (5)	0.0010 (6)
C2	0.0169 (6)	0.0207 (7)	0.0142 (7)	−0.0020 (5)	0.0033 (5)	−0.0001 (6)
C3	0.0147 (6)	0.0165 (7)	0.0194 (7)	−0.0025 (5)	0.0027 (5)	−0.0030 (6)
C4	0.0184 (6)	0.0165 (7)	0.0196 (7)	−0.0003 (5)	0.0021 (5)	0.0027 (6)
C5	0.0179 (7)	0.0221 (8)	0.0139 (7)	−0.0035 (6)	0.0039 (5)	0.0001 (5)
C6	0.0135 (6)	0.0182 (7)	0.0182 (7)	−0.0028 (5)	0.0036 (5)	−0.0020 (6)
C7	0.0141 (6)	0.0212 (7)	0.0183 (7)	−0.0040 (5)	0.0029 (5)	−0.0021 (6)
C8	0.0158 (6)	0.0166 (7)	0.0167 (7)	−0.0056 (5)	0.0039 (5)	−0.0020 (6)
C9	0.0135 (6)	0.0163 (7)	0.0169 (7)	−0.0038 (5)	0.0016 (5)	−0.0029 (5)
C10	0.0178 (6)	0.0162 (7)	0.0172 (7)	−0.0016 (5)	0.0023 (5)	0.0004 (5)
C11	0.0171 (6)	0.0220 (7)	0.0147 (7)	−0.0029 (6)	0.0033 (5)	−0.0014 (6)
C12	0.0134 (6)	0.0193 (7)	0.0185 (7)	−0.0008 (5)	0.0023 (5)	−0.0062 (6)
C13	0.0180 (7)	0.0180 (7)	0.0183 (7)	−0.0006 (6)	−0.0005 (5)	−0.0009 (6)
C14	0.0171 (7)	0.0208 (7)	0.0146 (7)	−0.0030 (5)	0.0025 (5)	−0.0008 (6)

### Geometric parameters (Å, °)

**Table:**

O1—C1	1.3537 (17)	C4—C5	1.3729 (19)
O1—H1	0.860 (15)	C4—H4	0.9500
O2—C3	1.3576 (16)	C5—C6	1.409 (2)
O2—H2A	0.839 (14)	C5—H5	0.9500
O3—C8	1.2336 (16)	C6—C7	1.4454 (19)
O4—N3	1.2196 (16)	C7—H7	0.9500
O5—N3	1.2337 (15)	C8—C9	1.4999 (18)
N1—C7	1.2868 (19)	C9—C14	1.3927 (19)
N1—N2	1.3884 (16)	C9—C10	1.3939 (19)
N2—C8	1.3441 (19)	C10—C11	1.3847 (19)
N2—H2B	0.865 (13)	C10—H10	0.9500
N3—C12	1.4705 (17)	C11—C12	1.384 (2)
C1—C2	1.3880 (19)	C11—H11	0.9500
C1—C6	1.4121 (19)	C12—C13	1.3825 (19)
C2—C3	1.389 (2)	C13—C14	1.3817 (19)
C2—H2	0.9500	C13—H13	0.9500
C3—C4	1.3996 (19)	C14—H14	0.9500
			
C1—O1—H1	108.8 (13)	C1—C6—C7	123.09 (13)
C3—O2—H2A	108.2 (12)	N1—C7—C6	121.51 (13)
C7—N1—N2	116.19 (12)	N1—C7—H7	119.2
C8—N2—N1	118.83 (12)	C6—C7—H7	119.2
C8—N2—H2B	126.4 (11)	O3—C8—N2	122.48 (12)
N1—N2—H2B	114.7 (11)	O3—C8—C9	119.76 (13)
O4—N3—O5	123.19 (12)	N2—C8—C9	117.77 (12)
O4—N3—C12	118.79 (12)	C14—C9—C10	119.77 (12)
O5—N3—C12	118.00 (12)	C14—C9—C8	115.90 (12)
O1—C1—C2	116.57 (12)	C10—C9—C8	124.31 (13)
O1—C1—C6	122.64 (12)	C11—C10—C9	120.27 (13)
C2—C1—C6	120.78 (13)	C11—C10—H10	119.9
C1—C2—C3	119.94 (13)	C9—C10—H10	119.9
C1—C2—H2	120.0	C12—C11—C10	118.33 (13)
C3—C2—H2	120.0	C12—C11—H11	120.8
O2—C3—C2	122.02 (12)	C10—C11—H11	120.8
O2—C3—C4	117.61 (12)	C13—C12—C11	122.80 (13)
C2—C3—C4	120.36 (12)	C13—C12—N3	118.07 (13)
C5—C4—C3	119.47 (13)	C11—C12—N3	119.12 (12)
C5—C4—H4	120.3	C14—C13—C12	118.11 (13)
C3—C4—H4	120.3	C14—C13—H13	120.9
C4—C5—C6	121.79 (13)	C12—C13—H13	120.9
C4—C5—H5	119.1	C13—C14—C9	120.69 (13)
C6—C5—H5	119.1	C13—C14—H14	119.7
C5—C6—C1	117.65 (12)	C9—C14—H14	119.7
C5—C6—C7	119.27 (13)		

### Hydrogen-bond geometry (Å, °)

**Table:**

*D*—H···*A*	*D*—H	H···*A*	*D*···*A*	*D*—H···*A*
O1—H1···N1	0.86 (2)	1.93 (2)	2.6818 (15)	146.(2)
O2—H2A···O3^i^	0.84 (1)	1.84 (2)	2.6759 (14)	173.(2)
N2—H2B···O5^ii^	0.87 (1)	2.28 (1)	3.0606 (16)	150.(1)
C2—H2···O3^i^	0.95	2.47	3.1730 (17)	131
C4—H4···O4^iii^	0.95	2.54	3.3428 (17)	143
C7—H7···O5^ii^	0.95	2.40	3.2082 (17)	143
C10—H10···O4^ii^	0.95	2.52	3.3627 (18)	147

Symmetry codes: (i) −*x*, *y*+1/2, −*z*+5/2; (ii) −*x*+1, *y*+1/2, −*z*+3/2; (iii) *x*−1, *y*+1, *z*.
